# The Value of Routinely Collected Data in Evaluating Home Assessment and Modification Interventions to Prevent Falls in Older People: Systematic Literature Review

**DOI:** 10.2196/24728

**Published:** 2021-04-23

**Authors:** Helen Daniels, Joe Hollinghurst, Richard Fry, Andrew Clegg, Sarah Hillcoat-Nallétamby, Silviya Nikolova, Sarah E Rodgers, Neil Williams, Ashley Akbari

**Affiliations:** 1 Population Data Science Swansea University Swansea United Kingdom; 2 Academic Unit of Elderly Care and Rehabilitation University of Leeds Leeds United Kingdom; 3 Centre for Ageing and Dementia Research Swansea University Swansea United Kingdom; 4 Leeds Institute of Health Sciences Swansea University Leeds United Kingdom; 5 Public Health, Policy & Systems University of Liverpool Liverpool United Kingdom; 6 Care and Repair Cymru Cardiff United Kingdom

**Keywords:** falls, aged, routinely collected data, evaluation research, systematic review

## Abstract

**Background:**

Falls in older people commonly occur at home. Home assessment and modification (HAM) interventions can be effective in reducing falls; however, there are some concerns over the validity of evaluation findings. Routinely collected data could improve the quality of HAM evaluations and strengthen their evidence base.

**Objective:**

The aim of this study is to conduct a systematic review of the evidence of the use of routinely collected data in the evaluations of HAM interventions.

**Methods:**

We searched the following databases from inception until January 31, 2020: PubMed, Ovid, CINAHL, OpenGrey, CENTRAL, LILACS, and Web of Knowledge. Eligible studies were those evaluating HAMs designed to reduce falls involving participants aged 60 years or more. We included study protocols and full reports. Bias was assessed using the Risk Of Bias In Non-Randomized Studies of Interventions (ROBINS-I) tool.

**Results:**

A total of 7 eligible studies were identified in 8 papers. Government organizations provided the majority of data across studies, with health care providers and third-sector organizations also providing data. Studies used a range of demographic, clinical and health, and administrative data. The purpose of using routinely collected data spanned recruiting and creating a sample, stratification, generating independent variables or covariates, and measuring key study-related outcomes. Nonhome-based modification interventions (eg, in nursing homes) using routinely collected data were not included in this study. We included two protocols, which meant that the results of those studies were not available. MeSH headings were excluded from the PubMed search because of a reduction in specificity. This means that some studies that met the inclusion criteria may not have been identified.

**Conclusions:**

Routine data can be used successfully in many aspects of HAM evaluations and can reduce biases and improve other important design considerations. However, the use of these data in these studies is currently not widespread. There are a number of governance barriers to be overcome to allow these types of linkage and to ensure that the use of routinely collected data in evaluations of HAM interventions is exploited to its full potential.

## Introduction

### Background

Falls in older people are a major public health concern. In the United Kingdom, approximately 1 in 3 adults aged 65 years or more experience at least one fall a year, which can lead to serious injury, even death. Falls are the most common cause of death in this age group [1]. This situation is similar worldwide, where around 28%-35% of people in this age group fall every year; this increases to 32%-42% in those aged 70 years or more [2]. The effects of falls on a person can be devastating, not only physically but can result in a fear of falling in the future and a loss of confidence and independence and can have a significant impact on family, friends, and caregivers [3,4]. Annual National Health Service (NHS) expenditure on injurious falls is in excess of UK £2 billion (US $2.5 billion) [5]; in the United States, this figure amounted to US $50 billion in 2015 [6]. Health care costs per fall for older people in Finland and Australia are US $3611 and US $1049, respectively [2]. Furthermore, the costs of hospital, community, and social care continue to significantly accrue 12 months after a fall [1]. One of the main risk factors for falls is increasing age; the incidence of falls begins to rise beyond the age of 65 years [7]. Given that there is an additional 8.2 million people aged 65 years and more projected for the United Kingdom in 2050 [8], preventative measures for falls in this age group will be key to reducing costs [5].

International evidence suggests that falls in older people commonly occur at home (around 35% of people more than 65 years of age) [2,7,8], and these are associated with higher morbidity, earlier mortality, and health inequalities [5-9]. Hazards in the home are associated with injury, and by using risk assessments, home assessment and modification (HAM) interventions identify potential environmental hazards present in the home [10]. Measures are then agreed to reduce these, such as the removal of bath mats or inclusion of handrails on stairs [4]. A Cochrane review by Gillespie et al [11] found that HAM interventions were effective in reducing both the rate of falls and the risk of falling in older people. After gathering evidence on effectiveness, the National Institute for Health and Care Excellence [5] have recommended that all older people living in the community at an increased risk of falls should be considered for these interventions.

Issues surrounding the evaluation of these HAM interventions could call into question the validity of their findings. Past criticism of these trials has been over a lack of an adequate control group and rigorous design, that they are underpowered, and that follow-up times have been found to be lacking [12,13]. Most HAM trials included in the review by Gillespie et al [11] had follow-up times of 1 year or less; therefore, it is unclear whether the effects of these interventions could be sustained beyond this [11-13]. Trials often exclude participants with comorbid conditions, particularly cognitive impairment [14,15]; however, cognitive impairment is a risk factor for falls in older people [16]. This can threaten the generalizability of a trial’s findings. The research burden of extensive study assessments is a particular concern for this participant group and can cause study attrition [17]. Conversely, minimizing the amount of data collection to reduce this burden could limit the usefulness of a trial. Furthermore, different variables are needed to stratify participant groups and to control for confounders (eg, past falls, polypharmacy, and socioeconomic status) to determine for whom the intervention works best and why [18,19]. Finally, recall bias could cause errors in self-report data and skew evaluation results [20].

It has been suggested that the use of routinely collected data in aging-related research has the potential to improve research quality and efficiency. These large-scale data sources allow for larger sample sizes and, therefore, the possibility for stratifying the sample with respect to key covariates and allow for longer follow-up times and reduced study attrition, especially given the age profile of the target population. In addition, these data help build an understanding of fall patterns and of individual treatment pathways [21]. Data collected routinely from health and social care interactions are increasingly being used in research; electronic health records (EHRs) are a prime example of this. Such data can be highly useful in health research; its nonuse may even cause harm [22]. The use of routine data in HAM intervention evaluations may be particularly useful, as it could help address the many challenges identified above regarding participant characteristics and trial conduct. To date, we conducted a systematic review to investigate the use of routinely collected data in HAM intervention evaluations.

### Objectives

Our objective is to conduct a systematic review to identify research studies using routinely collected administrative and EHR data to evaluate HAM interventions whose primary purpose is to reduce falls in older people. Given the aforementioned problems with prospectively collected data, namely, (1) lack of adequate control group, (2) short follow-up times, (3) lack of diversity in participants leading to a lack of generalizability of results, (4) research burden on participants because of extensive data collection, (5) study attrition (especially because of point 4), (6) lack of rich data (especially at baseline that would allow stratification of participant groups), and (7) recall bias, our rationale for undertaking this study was to understand the extent of routine data use in this field as an alternative. We aim to summarize the types of routinely collected data and their sources and to identify the questions that these data can answer. We also investigated the different methods and approaches of using these data. Finally, we sought to highlight the benefits and limitations of using these data, compared with other data types, in the evaluation of HAM interventions.

## Methods

### Search Strategy

**Figure 1:**
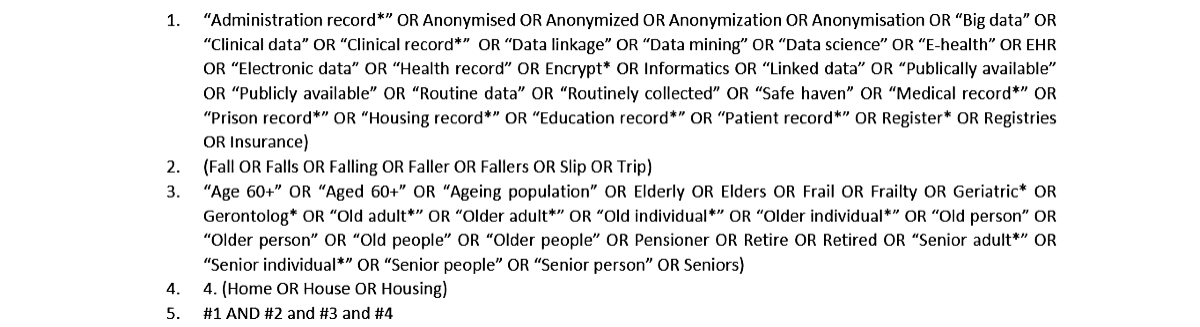
Search strategy for PubMed.

Review methods followed the University of York Centre for Reviews and Dissemination [23] guidance, and reporting followed the PRISMA (Preferred Reporting Items for Systematic Reviews and Meta-analyses) [24] guidelines where relevant (PRISMA checklist, Multimedia Appendix 1 [24]. To identify relevant studies, we searched the following databases from inception until January 31, 2020: PubMed, Ovid, CINAHL, OpenGrey, CENTRAL, LILACS, and Web of Knowledge. We used the keywords developed for a previous study designed to capture all types of routine data [25] and keywords to represent falls, older people, and the home. Figure 1 shows the search strategy used for PubMed, which was adapted for use with each database. Limits were abstract only. In addition, we searched the reference lists of potentially relevant papers and systematic reviews.

### Inclusion and Exclusion Criteria

We included primary research reporting HAM intervention evaluations that used routinely collected data, defined as data collected as a matter of course and not specifically for research [26]. We defined HAM as an assessment by a professional to identify environmental hazards and their removal or reduction by modifications to the home. The aim of these interventions must have been to reduce falls among older people living at home in the community. Studies including participants aged less than 60 years were excluded to ensure that these interventions were specifically targeted to the older population. Any control group was eligible because the effects of the intervention were not reviewed here. Potentially eligible studies included clinical effectiveness, cost-effectiveness, or process evaluations and could be of any study design. We included original research and study protocols (if a description of the full study was not yet published) from peer-reviewed journals, conference proceedings, clinical guidelines, and policy documents. Papers not written in the English language were included if an English translation of the abstract was available.

### Screening and Data Extraction

One reviewer screened the titles and abstracts of the papers identified by the search using the aforementioned criteria. Full-text papers were retrieved if deemed potentially eligible and assessed by two independent reviewers. A third reviewer was able to resolve any disagreements. We used a piloted and standardized data extraction form and contacted the study authors for additional information where needed.

### Risk of Bias

We assessed the risk of bias using the Risk Of Bias In Non-Randomized Studies of Interventions (ROBINS-I) tool [27]. The Cochrane Methods Bias Group [28] recommends this tool for use in systematic reviews that include nonrandomized controlled studies, and this tool allowed us to assess bias consistently across all our included studies, regardless of their study design. The tool covers seven domains that correspond to the risk of bias that can arise from different aspects of a study. For each domain, there are a number of questions to answer that will indicate whether this risk is low, moderate, serious, or critical or that there is not enough information to make a judgment. From these, following the tool’s guidance, an overall judgment about the risk of bias can then be reached. We identified studies’ main sources of bias in these domains, while focusing on the number or rate of falls as the main outcome of interest. We also looked for any other potential sources of bias or study design issues, paying particular attention to bias arising from the types of data used.

### Data Synthesis and Organization

Given the heterogeneity between studies’ interventions, participants, and other factors, a meta-analysis was not appropriate. Instead, we summarized the findings using narrative synthesis organized into 3 broad areas:

1. The source of routine data, aligned with the 3 main sources of routinely collected data used in health research (health care providers, government agencies, and nongovernmental and third-sector organizations)
2. The type of routine data, including demographic data relating to the characteristics of a person and where they live, clinical and health data generated by a clinical encounter or relating to a person’s health or health care, and administrative data gathered during the running of organizations (eg, registering people, for record-keeping, or when delivering a service)
3. The purpose of routine data, with 4 main categories: recruitment of participants and creation of study sample; stratification of the sample, a technique used to ensure that there is equal representation of a particular characteristic (eg, sex) or to enable subgroup analyses; generation of predictor variables and other covariates to measure effect on evaluation outcomes; and generation of outcome measures used to evaluate the effectiveness of the HAM interventions [29].

## Results

### Overview

**Figure 2:**
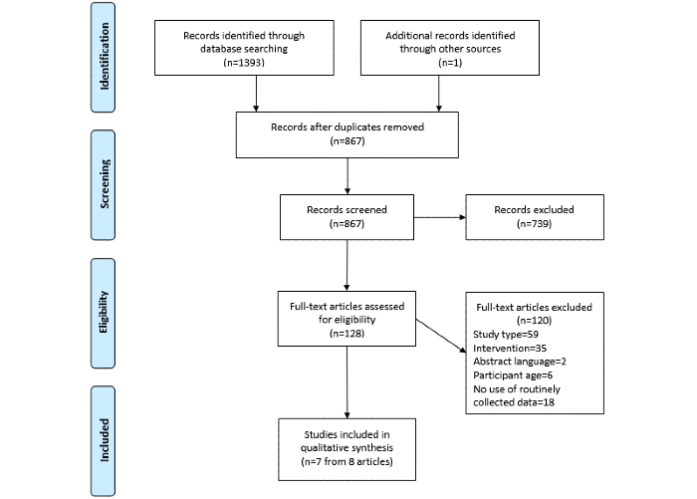
PRISMA (Preferred Reporting Items for Systematic Reviews and Meta-analyses) flow diagram.

After removing duplicates, we identified 867 papers in total—866 abstracts using electronic databases and 1 additional paper from the reference lists of the included studies. A total of 128 papers were identified as potentially eligible, and full-text papers were retrieved. Eight papers reporting 7 different studies met the inclusion criteria and were included in this study. All the studies were written in English. Two were protocol papers [18,30], and the remainder were reports of evaluations. Figure 2 summarizes the flow of studies in a PRISMA diagram [31].

Multimedia Appendix 2 [14,18,30,32-36] summarizes the details of the studies included in this review. Each of these studies evaluated HAM interventions, either for effectiveness or for cost-effectiveness, and consisted of home assessment by an occupational therapist or other trained personnel in addition to the removal or adaption of potential hazards. Of the 7 papers, 5 used randomized controlled trial designs, whereas the studies by Hollinghurst et al [18], de Almeida Mello et al [32], and Maggi et al [33] used a longitudinal quasi-experimental design. The research was located in the following countries: Australia [14,34], Belgium [32,33], the United Kingdom [18]), New Zealand [35,36], and the United States [30].

### Sources of Routine Data

The sources of routine data across all studies are summarized in Multimedia Appendix 2 For data sources hosting multiple databases, this table notes the original sources of each of these databases.

#### Health Care Providers

Two studies in this study used data from health care providers: in New Zealand—the University of Auckland optometry clinic, a private ophthalmology practice, and Dunedin and Auckland hospital [35], and in Australia—the Royal Prince Alfred Teaching and Research Hospital, Sydney [34]. The use of these data was approved by the Otago and Auckland ethics committees [35] and the Ethics Review Committee of the Central Sydney Area Health Service [34,37].

#### Government Agencies

Pega et al [36] used routine data obtained from the New Zealand Government’s Integrated Data Infrastructure (IDI) database [38]. This is a large database containing data on people and households in New Zealand, including data on education, income, benefits, migration, justice, and health. Many data sets within the IDI can be electronically linked using identifiable data—first and last name, date of birth, age, sex, and country of birth, which are then removed or encrypted before their use in research. The IDI follows strict governance procedures to ensure privacy and confidentially, including the use of a virtual platform to provide researchers access to data. Before accessing data, researchers must undertake a two-stage application process that costs US $500 plus tax (there is no charge for government organizations) [36,38].

The study reported by Maggi et al [33] and de Almeida Mello et al [32] sourced their routinely collected data from the Belgian Government’s InterMutualist Agency (IMA). This organization collects data on patients from Belgium’s 7 mutualities (health insurance associations) and prepares them for analysis [39]. Health insurance is mandatory in Belgium; therefore, it includes data on all legal residents (11 million citizens) [40]. The three main IMA databases include a population database of sociodemographic data, a health care database about health care utilization and cost data of ambulatory and hospital care, and a pharmaceutical database of medication prescription and cost data. These can be linked using multiple encrypted social security numbers [40]. Research use of person-level, pseudonomized health data requires an internal application and approval by the Information Security Committee and the supervision of a doctor, and access is provided via a virtual environment. This comes at a flat rate of €4660 or more if an analyst or medical expert is needed [39].

Day et al [14] used two official sources of data collected by the Australian Government. The electoral roll is managed by the Australian Electoral Commission [41] and under the *Commonwealth Electoral Act 1918,* which can be provided to approved medical researchers. The Australian Bureau of Statistics [42], a governmental organization that provides microdata to researchers and academics, curates the Australian national census and health survey. Recently, this organization has established DataLab, which is a web environment that gives users their own virtual workspace to access data where outputs can be vetted for disclosure risk. Access is given to accredited researchers only (the application process is outlined in detail [43]).

The St. Louis Area Agency on Aging (SLAAA) is a government organization that provides services and support for older people, including the HAM interventions evaluated by the Stark et al [30] study included in this paper [44]. The SLAAA collects data via the National Aging Program Information System database on the general health, nutritional, financial, functional, and environmental status of older adults in the area, and Stark et al [30] used these data as part of their study.

#### Nongovernmental or Third-Sector Organizations

Hollinghurst et al [18] used routinely collected data from the Secure Anonymized Information Linkage (SAIL) Databank [45]. SAIL is a data safe haven that houses many deidentified data sources predominantly about the Welsh population, including data from the NHS and the Welsh Government. Data sources in SAIL can be linked using twice-encrypted Anonymous Linking Fields based on a person’s NHS number or Residential Anonymous Linking Field for a place of residence derived from Unique Property Reference Numbers [46,47]. Technical and procedural controls such as an external Information Governance Review Panel (IGRP) and scrutiny of results by a SAIL Data Guardian mitigate the risk of disclosure. There is no charge for the data, except for support and infrastructure costs, such as data preparation and the use of computing [45,48]. Hollinghurst et al [18] accessed each of their data sources via the SAIL Databank.

Care and Repair Cymru [49] is a registered charity in the United Kingdom that provides HAM interventions to older people. They supplied Hollinghurst et al [18] study with data from their national registry, outlining information on their interventions and clients. Campbell et al [35] used data from the charity register of the Royal New Zealand Foundation for the Blind—a register of people who are living with vision loss in New Zealand.

### Types of Routine Data

#### Demographic Data

The most commonly used demographic data used by studies were age, date of birth, sex, and address or area of residence. Examples of the latter are lower layer super output areas (LSOAs), used in the study by Hollinghurst et al [18], which are small geographical areas consisting of 1000 to 1500 people in the United Kingdom. These data formed part of the Welsh Demographic Service Data Set, an NHS data source available in SAIL Databank [45] that gives the demographic characteristics of people registered with General Practitioner practices in Wales [18,50].

In addition to the main demographic data given earlier, Pega et al [36] obtained information on participants’ ethnicity and residential status from a database held by the New Zealand IDI database—the 2013 New Zealand Census of Population and Dwellings [38]. From the Australian national census and health survey, Day et al [14] extracted data on marital status, ethnicity, and type of residence (eg, own home or residential care). De Almeida Mello et al [32] and Maggi et al [33] used data on their participants’ financial situation and their cohabitants from the IMA’s database on reimbursed health care in Belgium.

#### Clinical and Health Data

Hollinghurst et al [18] used primary care data on patients’ symptoms, signs, diseases, disabilities, and abnormal laboratory values from the Welsh Longitudinal General Practice data in the SAIL Databank [45]. Most of these variables were in Read code format (Read version 2)—standardized clinical codes used by health professionals in the NHS to record patient data electronically [51]. Maggi et al [33] and de Almeida Mello et al [32] used primary care data from the Belgian IMA [39] database on medication use and information on the presence of a caregiver and the level of nursing care received by their participants.

Pega et al [36] accessed two other New Zealand IDI data sources to retrieve secondary care data from hospital events: the Ministry of Health’s New Zealand Health Tracker [52], which links data from primary and secondary care pertaining to publicly funded health events, and the Accident Compensation Corporation claims register, which contains information on all people to whom they have provided compensation for a nonfault accident. These data included the date of hospital admission, discharge, and type of admission, as indicated by the International Classification of Disease, version 10 (ICD-10) coding. ICD-10 is an international classification system for diseases, causes of injury, signs and symptoms, and social circumstances [53]. Campbell et al [35], Hollinghurst et al [18], and Salkeld et al [34] used similar data. ICD-10 codes were also used by studies to ascertain the cause of a participant’s death (if relevant) along with the date the death occurred; these data were extracted from the Belgian IMA database (de Almeida Mello et al [32] and Maggi et al [33]), and from the Welsh Annual District Death Extract, a government-curated register of all deaths relating to residents of Wales [18,53].

#### Administrative Data

Day et al [14] used self-report administrative data regarding the health status of the Australian population and their consumption of antidepressant and hypnotic medication collected as part of the Australian census and health survey. From the US St. Louis Area Agency on Aging NAPIS database, Stark et al [30] used self-report data about participants’ previous falls and fear of falling. This government organization keeps a record of this information to help tailor their services to older people [44]. From data generated as part of their service provision in Wales, Care and Repair Cymru provided Hollinghurst et al [18] with information on HAM intervention types (eg, advice visit and stair rail) and their installation date.

Salkeld et al [34] included costs of hospital events in their study, recorded using Australian diagnosis-related groups. This Australian classification system is related to the number and types of patients treated in a hospital. Currently known as Australian refined diagnosis-related groups, each group has a cost attached and is used for hospital economic analyses [54]. Health Resource Group codes are the UK equivalent of these and were used by Hollinghurst et al [18] from a secondary care data set called the Patient Episode Database for Wales. Maggi et al [33] and de Almeida Mello et al [32] also used resource utilization costs from the Belgian IMA database [39].

Every 4 to 5 years, the Welsh Government [55] calculates the Welsh Index of Multiple Deprivation from routine data, including income, employment, health, and education. Welsh Index of Multiple Deprivation ranks small areas in Wales (LSOAs) according to their level of deprivation (1-1909). Hollinghurst et al [18] used deprivation quintiles, which allocated deprivation rankings to each LSOA ranging from quintile 1 to quintile 5, where quintile 1 indicates the areas of highest deprivation. The Care Inspectorate Wales Care Home registry provided the addresses of all care settings in Wales who provide care services to the public [56]. Both these data sources were accessed via the SAIL Databank but are also publicly available online.

### Purpose of Routine Data

#### Recruitment of Participants or Creation of Study Sample

All but one of the studies included in this review used routine data to create study samples. Instead, Salkeld et al [34] recruited participants who were attending outpatient clinics and day centers for older people or were inpatients at a hospital.

Demographic and clinical information in routinely collected data allowed researchers to target their recruitment drive at participants according to their inclusion and exclusion criteria. Day et al [14] sent letters and made phone calls to 11,120 people aged over 70 years registered on the Australian electoral roll to recruit individuals who owned their own homes. Subsequently, researchers in this study used the Australian census and health survey to compare how their sample differed from the general population in terms of age, ethnicity, marital status, and health status [14]. Stark et al [30] formed their sample using SLAAA’s NAPIS database to identify individuals at high risk of falling (aged more than 65 years and having a fall in the preceding 12 months or worried about falling). Campbell et al [35] used data from the Royal New Zealand Foundation of the Blind register and clinic and hospital records to identify participants aged over 75 years with poor vision and living in the community. Eligible participants were then invited to participate. In the study reported by de Almeida Mello et al [32] and Maggi et al [33], the Belgian National Institute for Health and Disability Insurance that provided HAM interventions in the real world, gave records of those who had received HAM interventions so that researchers could recruit to their study.

Three of the studies reviewed created electronic cohorts from routine data. As these were formed from anonymized data sources, it was not necessary to seek participants’ consent to participate. Pega et al [36] used census data to identify the study population of people more than 65 years of age living in private accommodation. Hollinghurst et al [18] used data from their intervention provider, Care and Repair Cymru, to define a deidentified, electronic cohort of people living in Wales aged 60 years or more who received an intervention between 2009 and 2017. They also created a comparator group of people with similar demographic characteristics from the Welsh Longitudinal General Practice data who had not received an intervention from Care and Repair. Dates of death from the Annual District Death Extract from the Office of National Statistics [56] mortality data were used to censor participants who died during the course of the study. Maggi et al [33] and de Almeida Mello et al [32] also created an electronic cohort, this time for their comparator group only, from the Belgian IMA database. Using variables in the National Health Insurance data held in this database, researchers ensured that their intervention and comparator groups were matched in terms of age, risk of institutionalization, and health care utilization.

#### Stratification of the Sample

Pega et al [36] used census data to stratify their sample into 20 discrete cohorts by sex, age (65-69, 70-74, 75-79, 80-84, and 85 years or more), ethnicity (Indigenous New Zealanders: Maori and non-Maori), and whether participants were at high or low risk. The latter was determined according to the occurrence of any injurious falls in the previous five years identified using CD10. Maggi et al [33] de Almeida Mello et al [32] used routine data from the Belgian IMA [39] database to stratify their comparator group according to a participant’s health impairment (mild or moderate to severe). This variable was derived from information on age, the cost of nursing, physiotherapy, and speech therapy at home, type of nursing care, and use of drugs for dementia.

Hollinghurst et al [18] stratified their sample according to participants’ electronic Frailty Index (eFI) score. This index is used to predict outcomes including mortality, unplanned hospitalization, and nursing home admission and was calculated from 36 health deficit variables routinely collected and recorded in the Wales Longitudinal General Practice data set [57,58]. Depending on their eFI score, participants were categorized as either fit, mildly frail, moderately frail, or severely frail.

#### Generation of Predictor Variables or Covariates

Through electronic linkage between data sources within the SAIL Databank [45], Hollinghurst et al [18] assigned a deprivation index to the LSOA of participants’ residences to explore whether deprivation levels, together with age and sex, modify the effectiveness of HAM interventions. Pega et al [36] used census data to create a variable indicating the proportion of people moving house at or after 65 years of age to estimate transitions into and out of modified and unmodified accommodation and to calculate the probability of participants moving into residential care.

#### Generation of Outcome Measures

To investigate whether their HAM intervention was cost-effective, Pega et al [36] compared the cost of hospitalization and the cost of attending a nonhospital health care setting after a fall between groups. As a secondary outcome measure, this study calculated the probability of hospitalization after a fall. To assess the cost-effectiveness of their HAM intervention, Salkeld et al [34] calculated the cost of participants’ hospital utilization from the number of bed days and associated Australian diagnosis-related groups for each stay. Missing codes were imputed using the daily cost averaged across all codes. Campbell et al [35] measured the effectiveness of their HAM intervention according to the incidence of falls occurring postintervention using self-report calendars. Any falls reported as needing medical attention were confirmed using routine clinical data from hospitals and general practice records.

Maggi et al [33] and de Almeida Mello et al [32] linked their study participants individually to the IMA (2020) database, and this provided the main outcomes for their study; permanent institutionalization is defined as 90+ days at a nursing home or death. Individual-to-household data linkage allowed Hollinghurst et al [18] to measure intervention effectiveness from the number of hospital admissions for falls at home (identified with an ICD10 code) and the length of stay derived from admission and discharge dates. This study also measured the time it took for an individual to move to a care home after a fall using an anonymized list of care home addresses (from the Care Inspectorate Wales registry) to address changes in the Welsh Demographic Service data.

### Risk of Bias Findings

Table 1 shows the decisions regarding the risk of bias in each domain of the ROBINS-I tool and the decision made regarding the risk of bias in each study overall. After assessing the risk of bias, we judged that the studies included in this study were at an overall risk of low [14-36] to moderate bias [18,30,32,33]. The differences in bias were mainly because of design—all studies with overall low bias were randomized controlled trials (RCTs). The studies by Hollinghurst et al [18], de Almeida Mello et al [32], and Maggi et al [33] were longitudinal studies and, as such, were unable to reduce biases in the way that RCTs are inherently designed to do. Hollinghurst et al [18] did not adjust for previous falls, which is a predictor of subsequent falls; therefore, it was deemed at a serious risk of bias for confounding. The study by Stark et al [30] was a protocol only; therefore, there was not enough information available to determine whether this RCT was an overall low risk of bias.

It is worth mentioning that in the context of this study, using routinely collected data to measure outcomes can reduce the likelihood of bias in this domain. Blinding of participants is not possible in HAM evaluations; studies relying on self-report data to measure the number of falls are therefore subject to at least moderate bias (also known as response bias [29,30,32-34]. Both Hollinghurst et al [18] and Campbell et al [35] retrieved the number of falls from EHRs and were thus judged to be at a low risk of bias. Pega et al [36] also used routinely collected data to measure outcomes (New Zealand Tracker and Accident Compensation Corporation claims register). However, these two sources of data were not individually linked, and duplicated counts of falls may have occurred in some cases. This study was deemed to have a moderate risk of bias.

Recall bias occurs when participants misremember previous events or experiences and can lead to inaccuracies in the information recorded in studies [59]. As mentioned earlier, several studies used self-report data to account for previous falls, an important confounding factor, and inaccurate recall could result in an imbalance between groups, particularly in nonrandomized studies. For the studies that collected information on previous falls, only Pega et al [36] used routinely collected data to identify participants who had previously fallen, thus avoiding recall bias in this instance. Self-reported outcomes are particularly subject to this type of bias, where preexisting beliefs and memory can affect recall and sway study results in either direction [59]. In this study, four studies measured falls using self-report data and were, therefore, at risk of recall bias.

**Table 1:** Results of risk of bias assessment using the Risk Of Bias In Non-Randomized Studies of Interventions tool.

Reference	Domain								Overall risk
	Confounding	Selection bias	Classification bias	Deviation bias	Missing data	Outcome bias	Selective reporting		
Campbell et al [35]	Low	Low	Low	Moderate	Low	Moderate	Low	Low	
Day et al [14]	Low	Low	Low	Moderate	Low	Moderate	Low	Low	
de Almeida Mello et al [32] and Maggi et al [33]	Moderate	Low	Low	Low	Moderate	Moderate	Moderate	Moderate	
Hollinghurst et al [18]	Serious	Low	Low	Moderate	Low	Low	No information	Moderate	
Pega et al [36]	Low	Low	No information	Low	Low	Moderate	Moderate	Low	
Salkeld et al [34]	Low	Low	Low	Low	Low	Moderate	Low	Low	
Stark et al [30]	Low	Low	Low	No information	No information	Moderate	No information	Moderate	

Sampling bias, also known as volunteer bias, occurs when participants consist of individuals who have volunteered to participate in a study and may not be representative of the general population [59]. Only the study by Hollinghurst et al [18] was able to avoid this bias completely by using an anonymized electronic cohort, which precluded the need to seek participants’ consent to participate.

Small sample sizes in intervention evaluations are problematic as they are often unable to detect significant or clinically relevant differences, and findings cannot be extrapolated to the general population [60]. After conducting a power calculation to determine their target sample size, Salkeld et al [34] reported that their study of 530 participants was underpowered and the difference in the number of falls between groups was not significant. This may have been a type II error rather than a true reflection of the effectiveness of HAM interventions [37]. Failing to hit sample size targets was likely related to their recruitment strategy (as mentioned earlier) and budget constraints [34]. All other studies in this study used routine data to recruit participants and reported no issues regarding sample size. Studies that featured electronic cohorts were over 8 times larger than those using recruitment models requiring consent [18,32,33,36].

Limited follow-up times can also be an issue in trials, as any long-term hazards or benefits can be missed and are usually only able to provide evidence of effectiveness in the short term [61]. Using self-report outcomes for falls, Maggi et al [33] conducted a 6-month follow-up—the shortest follow-up time for studies in this paper. The longest was in the study by Pega et al [36] that used routinely collected data to follow up outcomes until a participant’s death or until age 110 years, although this was simulated data used in Markov modeling.

## Discussion

### Principal Findings

We identified 7 studies reported in 8 papers that used routine data to support the evaluation of HAM interventions. All studies were conducted in economically developed countries [62]. Government organizations provided the majority of data across studies, with health care providers and third-sector organizations providing data. Studies used a range of demographic, clinical or health, and administrative data. The purpose of using routinely collected data spanned recruiting or creating a sample, stratification, generating independent variables or covariates, and measuring key study-related outcomes.

The use of clinical data in research, particularly from EHRs, has risen considerably over recent years, and these are likely the most widely used source of routine data in health research today [63]. This coincides with a global increase (46% during 2011-2016) in EHR adoption across all health care service providers [64]. In this study, we have seen the versatile use of EHRs in HAM evaluations, from recruitment to outcome measurement. Although the provision of data by governments for health research is not as widespread, it is increasingly being advocated given the richness of the data they collect [65-67]. This is evident from the varied use of governmental data by the studies of this paper. In addition, many charities are now recognizing the value of their data, not only to improve their own services but also to enhance health research more generally [68,69].

Although the use of routinely collected data in health research is increasing, we only identified 7 studies that matched our inclusion criteria. This could be due to governance and system barriers surrounding the use of routinely collected data and data linkage in particular [22], although data safe havens used in studies from Wales [18] and New Zealand [36] improve accessibility. Linkage of household-level data is currently not commonplace, and there is a need for this type of data and linkage of data for evaluation purposes more generally [21,70]. The absence of any studies from low-to-middle income countries may be a result of having fewer housing adaption provision in conjunction with less well-established routine data collection infrastructure and procedures [71,72].

This study shows that there is a clear value in the use of routinely collected data in HAM evaluation. It allows for objective data collection on key outcomes, including hospitalization, length of stay, care home admission, and mortality. Its use can reduce the risk of bias in trials where assessors may become unblinded to allocation and in participant recall and response. Longitudinal data sets such as EHRs are continuously updated and preclude the need for taking repeated measures from participants; this can reduce research costs and the burden on participants. In addition, target populations meeting specific inclusion criteria can be easily accessed. Forming electronic cohorts from routine data has particular benefits in that they can minimize recruitment bias and attrition, plus extended follow up and the creation of suitable control groups are far simpler to achieve. These larger samples also allow for greater validity, generalizability, and yield adequate statistical power. Moreover, hard-to-reach or minority populations that are typically underrepresented in research can be easily included, such as those of advanced old age and other underrepresented groups including minority ethnic groups and people with multimorbidities [36,73,74]. However, too large a sample can detect significant differences that are not clinically relevant, and care needs to be taken to ensure appropriate sample size calculation with predefined clinically important differences in outcomes where possible [60].

Routine data are often used in mixed methods studies. The variables collected in these data sets are predetermined and rigid, generally restricted to codes, and rarely contain any contextual or in-depth qualitative information. These studies still need qualitative data and process evaluation to understand how service users experience these interventions and to get to the core of what actually matters to older people—all of which can influence an intervention’s effectiveness [75]. Specific data collection via surveys and interviews is necessary to collect granular data or to answer specific questions that are not supported by routine data. If the use of routine data can reduce the burden of quantitative data collection, then more resources can be made available to enhance these data using alternative methods (Textbox 1) [76].

**Potential benefits and limitations of the use of routinely collected data in home assessment modification evaluations.**

**Benefits**

- Rich data
- No or minimal participant burden
- Reusable for replication studies
- High external validity
- Diverse populations
- Fewer requirements for ethical approval
- Large control groups available for adequate statistical power
- Avoids recall bias
- Reduces study attrition
- No interference with routine care
- Lower cost and less resources needed for data collection
- Long observation periods

**Limitations**

- Data quality, for example, missingness
- Outcomes may need to be derived
- Randomization studies not possible with use of these data only
- Unavailability of confounding variables
- Data access issues, for example, governance
- Overpowered studies leading to spurious, significant findings
- Participant contact often not possible with anonymized routine data as consent not sought at study inception

### Benefits and Limitations of This Study

Despite the use of rigorous methods, this study has several limitations. Some studies may not have fully reported the use of routine data in their publications; therefore, this study may be missing key information in this regard. We focused only on fall interventions based in participants’ homes; nonhome-based fall intervention evaluations using routinely collected data were excluded. Two protocols were included; therefore, results were not available for either of these studies. We did not attempt to identify unpublished studies, which means that this study could be subject to publication bias. We did not use MeSH headings in the PubMed search as, after piloting, this greatly reduced the search specificity. This means that some studies that met the inclusion criteria may not have been identified. However, we used a comprehensive search strategy, including the use of the LILACS database, of which most of its indexed journals are not indexed in other databases [77]. Our robust bias assessment using a standardized tool allowed objective evidence of bias in the studies of this paper and the comparison of bias between routine and nonroutine data use.

### Conclusions

Despite the limited number of studies, we have seen that routine data can be used successfully in many aspects of evaluations of HAMs and can enhance methodological quality by reducing different types of bias while also improving other important design considerations. These advantages could be used further, for example, in the evaluation of HAM interventions to support people with disabilities. However, this study shows the under use of routine data in this important area of work. There are a number of governance barriers to be overcome to allow these types of linkage, and more work should be done to take advantage of the value that routinely collected data can offer.

## Appendix

Multimedia Appendix 1 PRISMA (Preferred Reporting Items for Systematic Reviews and Meta-analyses) checklist.

Multimedia Appendix 2 Summary of findings.

## Abbreviations

ADR: Administrative Data Research
eFI: electronic Frailty Index
EHR: electronic health record
HAM: home assessment and modification
ICD10: International Classification of Disease, version 10
IDI: integrated data infrastructure
IGRP: Information Governance Review Panel
IMA: InterMutualist Agency
LSOA: lower layer super output area
NHS: National Health Service
NIHR: National Institute for Health Research Applied Research
PRISMA: Preferred Reporting Items for Systematic Reviews and Meta-analyses
RCT: randomized controlled trial
ROBINS-I: Risk Of Bias In Non-Randomized Studies of Interventions
SAIL: Secure Anonymized Information Linkage
SLAAA: St. Louis Area Agency on Aging
