# Supplementary material for: The Value of Routinely Collected Data in Evaluating Home Assessment and Modification Interventions to Prevent Falls in Older People: Systematic Literature Review
Source: JMIR Aging. 2021 Apr 23;4(2):e24728. doi: 10.2196/24728 (PMC8105762; doi:10.2196/24728)
Supplement: Multimedia Appendix 2 [file aging_v4i2e24728_app2.docx]

**Multimedia Appendix 2.** Summary of findings.

|  | Study details | | | | Database/set | | | Category of data | Purpose of routine data | | | |
| --- | --- | --- | --- | --- | --- | --- | --- | --- | --- | --- | --- | --- |
| Reference | Country | Type of study | Sample size | Follow up | Healthcare provider | Government | Non-governmental/Third sector organisation |  | Recruitment/forming a sample | Stratification | Independent variables/covariates | Outcome measures |
| Campbell et al [35] | New Zealand | Effectiveness and cost effectiveness RCT | 391 | 1 year | University of Auckland optometry clinic  Dunedin and Auckland low vision outpatient clinic  Private ophthalmology practice |  | Royal New Zealand Foundation of the Blind | Demographic | People over 75 with poor vision and living in the community |  |  |  |
|  |  |  |  |  | Dunedin and Auckland hospital and general practices |  |  | Clinical/health |  |  |  | Number of injurious falls |
| Day et al [14] | Australia | Effectiveness RCT | 1090 | 18 months |  | Australian electoral roll |  | Demographic  Administrative | People over 70 living in their own home |  |  |  |
|  |  |  |  |  |  | Census and health survey |  | Demographic  Clinical/health | Assessing generalisability of sample for age, marital status, ethnicity, and health status |  |  |  |
| de Almeida Mello [32]  Maggi [33] | Belgium | Longitudinal quasi-experimental effectiveness study | 8,240 | 6 months (for falls outcomes) |  | National health Insurance Database (CIN-IMA) |  | Demographic  Clinical/health | Comparator group of frail people over 65 | Health impairment |  | Permanent institutionalisation  Death |
|  |  |  |  |  | Belgian National Institute for Health and Disability Insurance |  |  |  | Identification of people receiving an intervention |  |  |  |
| Hollinghurst et al [18] | Wales, UK | Quasi-experimental effectiveness study | ~440,000 | 1-5 years | Welsh Longitudinal General Practice database |  |  | Clinical/health |  | Electronic Frailty Index |  |  |
|  |  |  |  |  | Patient Episode Database for Wales |  |  | Clinical health  Administrative |  |  |  | Number of hospital admissions for falls  Length of stay  Cost of stay |
|  |  |  |  |  | Emergency Department Data Set |  |  | Clinical/health |  |  |  |  |
|  |  |  |  |  | Welsh Demographic Service dataset |  |  | Demographic | People over 60 |  | Age, sex | Time to a care home after a fall |
|  |  |  |  |  |  | Care Inspectorate Wales Care Home registry |  | Administrative |  |  |  | Time to a care home after a fall |
|  |  |  |  |  |  | Annual District Death Extract |  | Clinical/health | Death, cause of death |  |  |  |
|  |  |  |  |  |  | Welsh Index of Deprivation |  | Administrative |  |  | Deprivation index of dwelling |  |
|  |  |  |  |  |  |  | Care and Repair national register | Administrative |  |  | Date and type of intervention |  |
| Pega et al [36] | New Zealand | Cost effectiveness cluster RCT | New Zealand population over 65 | Until death or participant age 110 years |  | 2013 New Zealand Census of Population and Dwellings |  | Demographic | People over 65 and living in private accommodation | Sex, age, ethnicity | Proportion of people moving house and/or into residential care |  |
|  |  |  |  |  |  | New Zealand Health Tracker hospitalisation register |  | Clinical/health |  | Occurrence of an injurious fall in the last 5 years |  | Probability of hospitalisation or death after falling  Falls in the previous five years |
|  |  |  |  |  |  | Accident Compensation Corporation claims register |  | Clinical/health |  |  |  | Probability of hospitalisation or death after falling  Falls in the previous five years |
| Salkeld et al [34] | Australia | Cost effectiveness RCT | 530 | 12 months | Royal Prince Alfred Hospital |  |  | Administrative  Clinical/health |  |  |  | Change in hospital utilisation cost |
| Stark et al [30] | USA | Effectiveness RCT | 300 | 12 months |  | St. Louis Area Agency on Aging |  | Demographic  Clinical/health | People over 65 at high risk of falling |  |  |  |
